# Scientific Items

**Published:** 1877-10-20

**Authors:** 


### Scientific Items.

Telephone.—A practical use for the telephone has been discovered by Dr. Foster, government inspector of mines in England. Dr. Foster sent a telephone down the ventilating shaft of the Eliza mine, at St. Austell, Cornwall, the instrument having been attached to a covered copper wire, and in less than fifteen minutes persons speaking at the bottom of the mine were distinctly audible on the surface. The telephone will supercede the inefficient cord signals hitherto used as a means of communication from the interior of deep mines to the ground above.—*Ex.*

Planet Mars.—The discovery of two satillites to the planet Mars occurred on the night of the 16th of August last, by Prof. Hall, at Washington. Mars bears a very strong resemblance to the earth, in many respects. Divisions of land and water are well defined; and that the seasons are like the earth's is evident from the alternate increase and decrease of the white snow caps about the poles. An atmosphere much like ours gives Mars its ruddy appearance, and bears clouds and storms that often obscure well-known features of its surface.

Locusts.—The seventeen year locusts appeared, the present summer, in certain Northern States. In the upper portion of Georgia they appear once in thirteen years. Their last appearance was in the spring of 1868, and hence may be looked for again in 1881

